# Analysis of the Objective and Subjective Stress Response of Students and Professors in Practical Nursing Exams and Their Relationship with Academic Performance

**DOI:** 10.3390/ijerph19159121

**Published:** 2022-07-26

**Authors:** Paula Sánchez-Conde, Ana Isabel Beltrán-Velasco, Vicente Javier Clemente-Suárez

**Affiliations:** 1 Faculty of Sports Sciences, Universidad Europea de Madrid, 28001 Madrid, Spain; pau.sanchez.conde@gmail.com (P.S.-C.); abeltranv@nebrija.es (A.I.B.-V.); 2 Department of Psychology, Faculty of Life and Natural Sciences, University of Nebrija, 28240 Madrid, Spain; 3 Grupo de Investigación en Cultura, Educación y Sociedad, Universidad de la Costa, Barranquilla 080001, Colombia

**Keywords:** stress response, autonomic modulation, grade, difficulty perception

## Abstract

The aim of the present research was to analyse the objective and subjective stress responses of students in a clinical case evaluation and the correlation with academic performance, as well as to analyse the differences in grade and difficulty perceptions between students and professors that designed the clinical case. A sample of 103 first-year students from a nursing degree was studied. In this sample, the objective stress was analysed by measuring the autonomic modulation (through the heart rate variability); moreover, the subjective stress was analysed using the SUDS scale. Furthermore, the difficulty perception and academic performance were measured using scales for both students and professors. The measures were taken before and after the clinical exams. A large subjective and objective stress response was observed at the beginning of the clinical case, and this response was related to a high academic performance perception. Upon completion of the clinical evaluation, both the stress response and the academic performance perception decreased. The professors and students presented different grade and difficulty perceptions concerning the clinical case.

## 1. Introduction

Within the European Higher Education Area, since the establishment of the Bologna Declaration (1999), academic focus has shifted to active learning and the student’s role as being responsible for their own skills and abilities for their professional future and career [1]. According to this new educational construct, new teaching and evaluation models have been introduced, such as clinical cases, simulations, the objective structured clinical evaluation (OSCE), seminars, etc. Practical learning is essential to strengthen acquired professional competencies and to promote engagement with students’ professional development [2]. Through the execution of clinical cases, students can apply acquired knowledge, make decisions, develop their critical thinking, and solve problems. These methods are also a novelty for professors, since they represent a new method of assessment, through continuous evaluation and not with a unique and final exam [3].

Training programs are critical for the acquisition of healthcare professionals’ competencies [4]. Specifically, future nursing professionals can learn and train their skills and abilities in a safe context without any risk to the patient through practical cases and clinical simulations [5]. Therefore, in a space resembling reality as much as possible, nursing students can train in many different techniques, communication skills, critical thinking, and teamwork, with the support, surveillance, and the immediate teaching of the professor in charge. This is helpful for students to acquire self-confidence and self-esteem while they incorporate their knowledge and attitudes [6].

Despite the great number of benefits of these new teaching methodologies that introduce the student to clinical practice, these are often perceived as stressors [7]. On the one hand, the student is forced to face unknown and uncontrollable situations, where they must develop the competencies and skills required for their future profession. On the other hand, the professor also perceives these situations as stressors, because they have to adapt different methods regarding the desired goals and identify the difficulty, stress, and motivation that students are experiencing in order to create a better educational stimulus [5]. This stress is expressed as a high sympathetic activation, and it is modulated by the autonomous nervous system [8]. Previous research has found how a high sympathetic activation interferes with the pre-frontal cortex connections of the brain, and how it has a negative impact on the memory and information processing [9], as well as with decision-making, concentrating capacity, and executive functions [10]. It has also been observed how stress can impact the brain’s plasticity and synaptic connections, interfering with learning and academic performance [11]. The sympathetic system activation can be measured using electronic devices through the analysis of the heart rate variability to assess the objective stress response by teachers [8]. This objective stress analysis has been widely used in different contexts, such as in the military [12], high-performance sports [13], and educational contexts [14].

Knowing the importance of active learning to manage the teaching process is essential to know the perception of the players involved, both the professors and students. In this line, being able to analyse students’ objective stress caused by this method of learning is essential to designing a correct educational context. For that reason, this research aimed to i. analyse the objective and subjective stress response of students in a clinical case evaluation and the correlation with academic performance and ii. analyse the differences in grade and difficulty perceptions between students and professors that designed the clinical case. The initial hypotheses were (i) a higher stress response would be related with lower academic performance and (ii) the difficulty and grade perceptions of the professors and students of the clinical evaluation would be different.

## 2. Materials and Methods

### 2.1. Participants

We analysed 103 first-year nursing degree students (81 females and 22 males, 20.1 ± 2.3 years). All the students had the same experience prior to facing the clinical practical evaluation; therefore, they all shared the same starting point and conditions regarding the acquisition of competences. The inclusion criteria were that they have no previous experience in clinical practice, that all students were in their first year of their nursing degree, and that all of them were conducting a clinical practical exam for the first time. The exclusion criteria were if participants took any drugs or if they had any previous clinical experience. All of the procedures were conducted following the Declaration of Helsinki (as revised in Brazil, 2013). All the participants filled out a consent form before starting the research and all the procedures were approved by the University Ethics Committee (CIPI/18/074).

### 2.2. Procedure

Clinical cases were set according to the students’ level of knowledge and were related to the theoretical knowledge provided. They lasted 10 min, and were conducted as follows: Students had to provide an answer to a clinical case that the professor gave to them. The clinical case was about a medical situation that consisted of some symptoms and the patient’s medical history. Students then had to think about a possible treatment and care plan.

### 2.3. Subjective Response Evaluation

We analysed the stress, grade, and difficulty perceptions of the professors at the end of the clinical case. We also analysed the stress and grade perception of students pre- and post-clinical case; we also measured the difficulty perception at the end of the clinical practice. Stress was measured on a subjective scale from 0 (no stress) to 100 (maximum stress), difficulty perception was measured from 0 (no difficulty) to 100 (maximum difficulty), and academic performance was measured on a 0 to 10 scale [5].

### 2.4. Objective Stress Response Evaluation

We recorded HRV at the beginning of the exam, at the first and second halves, using a validated device (Polar V800, Kempele, Finland) and analysed using the Kubios HRV software (version 2.0, Biosignal Analysis and Medical Imaging Group, University of Kuopio, Kuopio, Finland) [7]. The following HRV variables were analysed: HR: heart rate; PNN50: percentage of differences between normal adjacent R–R intervals greater than 50 ms; RMSSD: the square root of the average of the sum of differences squared between normal adjacent; LF: low frequency waves that were related to the sympathetic system; HF: high frequency waves that were related to the parasympathetic system; LF/HF ratio: low-frequency waves and high-frequency waves ratio; SD1: short-term variability of the HRV; SD2: long-term variability of the HRV.

### 2.5. Statistical Analysis

The SPSS statistical package (version 22.0; SPSS, Inc., Chicago, IL, USA) was used to analyse the data. The normality and homoscedasticity assumptions were checked with a Kolmogorov–Smirnov test. Differences between the pre- and post-grade stress perceptions of students were analysed using a *t*-test for dependent variables. Differences between students’ and professors’ grade and stress perceptions in the practical exam were analysed using a *t*-test for independent variables. Differences in HRV between the practical exam moments were analysed using a MANOVA with samples as a fixed factor and with a Bonferroni post hoc analysis. For this aim, the assumptions of sphericity and independence of data were confirmed with Mauchly’s test of sphericity and the Durbin–Watson contrast test, respectively. The effect size was tested using the partial η^2^. Finally, a Pearson bivariate correlation test was performed to analyse correlations between the grade of students in the practical exam and the subjective and objective stress (HRV) variables analysed. The level of significance for all the comparisons was set at *p* ≤ 0.05.

## 3. Results

Results are presented as mean ± standard deviation. We found a higher significant stress perception before developing the practice exam than after. We also observed a greater grade related to academic performance before the clinical exam than after taking it (Table 1).

The students showed a higher difficulty perception than the professors (Table 2). The bivariate correlation test between the grade of students in the practical exam and the subjective and objective stress (HRV) variables showed no significant correlations.

The HR was significantly lower prior to the exam (1) than in the 1° half (2) and the 2° half (3). RMMSD was higher prior to the exam (1) than in the 2° half (3), and the 1° half (2) was higher than the 2° half (3). PNN50 was higher prior to the exam (1) than in the 2° half (3), and the 1° half (2) was lower than the 2° half (3). SD1 was higher prior to the exam (1) than in the 2° half (3), and the 1° half (2) was lower than the 2° half (3). SD2 was lower in the 1° half (2) than in the 2° half (3) (Table 3).

Lastly, regarding the relationship between the mentioned variables, there was no significant correlation between the pre-evaluation stress, post-evaluation stress, and the HRV. A significant small negative correlation arose between the pre-evaluation stress and the grade perception before (r = −0.222; *p* = 0.024) and after (r = −0.324; *p* = 0.001) the evaluation. There was also a significant small positive correlation between the pre- and post-evaluation stress (r = 0.415; *p* = 0.000) and the difficulty perception (r = 0.371; *p* = 0.000). There was a small positive correlation of the post-stress with the difficulty perception (r = 0.282; *p* = 0.004) and a moderate negative correlation with the post-grade (r = −0.402; *p* = 0.000). Lastly, there was a small negative correlation between the difficulty perception and the post-grade (r = −0.279; *p* = 0.005).

## 4. Discussion

This research aimed to analyse the objective and subjective stress responses of nursing students in a clinical case evaluation, and the relationship with the students’ academic performance. Furthermore, it tried to find possible differences in grade and difficulty perceptions between students and the professors. The initial hypotheses were i. that a higher stress response would be related to lower academic performance, and that ii. the grade and difficulty perception of the professors and the students of the clinical evaluation would be different. With the obtained results, the first hypothesis could not be verified due to high levels of perceived stress being associated with a better perceived academic performance prior to the clinical case, and due to both of those parameters decreasing once the case was completed. This could also be because students with better academic performances were initially more focused on achieving the best possible result, so they had a higher level of stress during the exam. On the contrary, students with worse academic performance were less tuned in to the maximum result, and, therefore, their stress level was relatively low. Regarding the second hypothesis, it can be corroborated that since students and professors showed different perceptions of difficulty, as students perceived twice the difficulty than the professors. Additionally, different academic performance perceptions were also exhibited, with the professor’s ones being higher, but presenting smaller differences between them than the prior case.

The students showed a higher stress perception before undergoing the clinical case than once they completed it. This was due to the anticipatory anxiety that preluded facing an unknown event where the student had to put into practice the acquired knowledge, and, on top of that, they were going to be evaluated by someone else [7]. These results harmonised with previous studies on the subject, which found that 64.19% of students always or practically always exhibited stress before professors’ evaluations [15]. Other research pointed out that what worried a first-year nursing student the most was the lack of knowledge when facing a clinical situation [16], which is a factor that could increase the student’s stress perception in an evaluation situation. The present study showed how students became used to the stressful environment (evaluation), decreasing the stress perception upon the completion of the clinical case, though they still presented high levels of stress. This was due to the different appropriate coping strategies put in place, and due to the increase in self-confidence with exposure to the stressing factor, which was the clinical case. Previous research showed this stress habituation process in psychology students, in which the levels of stress decreased throughout a simulated therapy [17]. Something similar can also be seen with soldiers, where experience is related to a decrease in the stress response when facing determined situations [18]. This explains the results obtained in the present study, where certain habituation could be observed. However, this habituation was very discrete, as the present study was carried out with first-year students who did not possess enough experience to achieve greater habituation than that.

With regard to the academic performance perception, it can be seen how students expected to obtain higher academic grades before the clinical case than afterward, since the grade perception decreased. This could be explained due to the fact that, once the students were exposed to the clinical case, they have realised that their knowledge or skills were not enough to face the case successfully. Academic performance was , therefore, related to the self-perception and the self-appraisal of their capabilities. Green et al. (2006) confirm that by enhancing self-concept and motivation, the academic performance was also enhanced, and vice versa [19]. Moreover, academic performance was also related to the emotional intelligence and cognitive abilities of each student, the latter two being able to predict the expected performance [20]. Thus, at a cognitive level, it was observed that academic performance was influenced by motivation and learning strategies. At an emotional level, it was perceived that anxiety was another influential factor on the performance [21].

Students presented a difficulty perception higher than that of the professors’, which translated into the differences in the perception of academic performance; that is, students expected a worse performance than the one expected by the professors. These differences resided in the perception of the knowledge level prior to the test that each group underwent. On the one hand, students considered their education and learning process insufficient to face the case; therefore, they displayed a higher difficulty perception and a lower academic performance perception. These results highlight the importance of the teaching process and evaluation through competencies, where the students can acquire the abilities and skills necessary for their professional performance, and with that, they would feel more confident, alongside increasing their academic performance during their education. Furthermore, clinical simulations, case studies, etc., are fundamental for the learning process, as they can allow the student to become used to the clinical environment. This can decrease their difficulty perception through constant exposure, and with it the best adaptation to their future working context [22].

Previous studies have shown how the professor, who is responsible for the education and evaluation, considers the connection between what is suggested in the academic program and what is expected from the students once the evaluation methods are in place [23]. This would explain why the professor perceived a lower difficulty on the planned test, and why they expected a better academic performance than the students did. In addition, different causes of difficulties in the evaluation methods could be seen, in which the professor associated these difficulties with a lack of time and the number of students, whereas the students claimed that these difficulties were due to the lack of knowledge, professor-related factors, etc. [24].

Regarding the students’ objective stress, which was measured through the analysis of the heart rate variability, it was seen that the highest stress moment took place during the exposure to the stimulus, which was the students having to face the clinical case. This was observed by the increase in the HR and the decrease in the RMSSD and pNN50. Nevertheless, some other parameters, such as the LF, HF, and LF/HF ratio, did not show significant changes, and no changes were observed in the frequency domain parameters. At the end of the practical exam, a decrease in the stress response was observed due to the habituation process, represented by an increase in the RMSSD and the pNN50. The moment of greater objective stress was when the students had to put their knowledge and skills into practice and check whether they had sufficient preparation to overcome the clinical case proposed. This could be related to the so-called “evaluation anxiety”, where the perception of a situation as threatening can incur physiological, cognitive, and behavioural consequences [25]. After the case, it was observed that the signs of objective stress decreased, since the students were able to utilise the corresponding coping strategies and achieve a certain degree of adaptation to stress through repeated exposure. These results have also been observed in psychology students, where the sympathetic modulation and the subjective anxiety levels decreased once the clinical simulation was over [17]. Nonetheless, contradictory results were found in a study carried out with physiotherapy students. During the whole clinical practice, where the habituation process was absent, a high sustained sympathetic activity was registered [7]. The same case scenario was found in nursing students during the performance of an ECOE [5].

### Limitations of the Study and Future Research Lines

One of the limitations found during the present study was the lack of a measurement of the corresponding hormones (noradrenalin, cortisol, etc.) that were necessary for the stress response analysis. Future research could follow the aforementioned pathway to find out, in a more exhaustive way, the level of objective stress that students undergo.

Additionally, it would also be interesting for future investigations to compare different academic years, since, in this study, only first-year students were considered. It could be of high interest as well to compare the rankings and values across different fields of study within the health science environment and verify if there are any significant differences.

## 5. Conclusions

A large subjective and objective stress response was observed at the beginning of the clinical case, and this response was related to a high academic performance perception. Upon completion of the clinical evaluation, both the stress response and the academic performance perception decreased. The professors and students presented different grades and difficulty perceptions concerning the clinical case.

## Figures and Tables

**Table 1:** Subjective stress perception and grade perception differences before and after the practical exam.

					95% Confidence Interval of the Difference	
	Pre	Post	T	*p*	Lower	Upper
Grade (0–10)	6.21 ± 0.98	5.48 ± 1.40	5.57	0.00	0.47	0.99
Stress (0–100)	74.89 ± 18.89	62.02 ± 27.25	5.03	0.00	7.79	17.93

**Table 2:** Practical exam difficulty perception and grade differences between the students and professors.

					95% Confidence Interval of the Difference	
	Student	Professor	T	*p*	Lower	Upper
Difficulty (0–100)	60.37 ± 18.25	30.00 ± 0.00	16.80	0.000	26.79	33.96
Grade (0–10)	6.21 ± 0.98	7.67 ± 0.97	11.51	0.000	1.20	1.70

**Table 3:** Heart rate variability results from the practical exam. Mean HR: heart rate average; RMSSD: square root of the average of sum of the squared differences of the RR intervals; PNN50: percentage of consecutive RR intervals that differed >50 ms; LF: low-frequency waves; HF: high-frequency waves; LF/HF RATIO: rate between the low- and high-frequency waves; SD1: variability of the short-term HRV; SD2: variability of the long-term HRV.

	Pre (1)	1° Half (2)	2° Half (3)	F	*p*	η_p_^2^	Moment Comparison
HR	102.41 ± 14.17	111.01 ± 15.42	106.53 ± 14.69	43.96	0.00	0.48	1 < 3
							2 > 1
							2 > 3
RMMSD	35.64 ± 28.40	29.45 ± 20.78	32.71 ± 19.27	6.26	0.00	0.12	1 > 3
							2 < 3
PNN50	9.84 ± 11.06	7.26 ± 8.80	7.93 ± 8.78	6.40	0.00	0.12	1 > 3
							2 < 3
LF	74.48 ± 11.91	73.62 ± 12.29	74.89 ± 15.57	0.50	0.60	0.01	1 > 2
							1 < 3
							2 < 3
HF	25.43 ± 11.87	26.29 ± 12.24	25.02 ± 15.53	0.50	0.60	0.01	1 < 2
							1 > 3
							2 > 3
LF/HF ratio	5.89 ± 19.83	3.71 ± 2.23	3.01 + 1.93	1.69	0.19	0.03	1 > 2
							1 < 3
							2 < 3
SD1	25.22 ± 20.09	20.84 ± 14.72	23.15 ± 13.64	6.25	0.00	0.12	1 > 3
							2 < 3
SD2	102.63 ± 45.50	88.47 ± 48.30	90.08 ± 49.83	5.73	0.00	0.11	2 < 3

## Data Availability

All data are included in the manuscript.
